# Localized Intestinal Radiation and Liquid Diet Enhance Survival and Permit Evaluation of Long-Term Intestinal Responses to High Dose Radiation in Mice

**DOI:** 10.1371/journal.pone.0051310

**Published:** 2012-12-07

**Authors:** Laurianne Van Landeghem, Randall Eric Blue, Jeffrey J. Dehmer, Susan J. Henning, Michael A. Helmrath, Pauline Kay Lund

**Affiliations:** 1 Department of Cell Biology and Physiology, University of North Carolina, Chapel Hill, North Carolina, United States of America; 2 Department of Surgery, University of North Carolina, Chapel Hill, North Carolina, United States of America; 3 Department of Medicine, University of North Carolina, Chapel Hill, North Carolina, United States of America; 4 Department of Surgery, University of Cincinnati, Cincinnati, Ohio, United States of America; Stem Cell Research Institute, Belgium

## Abstract

**Background:**

*In vivo* studies of high dose radiation-induced crypt and intestinal stem cell (ISC) loss and subsequent regeneration are typically restricted to 5–8 days after radiation due to high mortality and immune failure. This study aimed to develop murine radiation models of complete crypt loss that permit longer-term studies of ISC and crypt regeneration, repair and normalization of the intestinal epithelium.

**Methods:**

In C57Bl/6J mice, a predetermined small intestinal segment was exteriorized and exposed to 14Gy-radiation, while a lead shield protected the rest of the body from radiation. Sham controls had segment exteriorization but no radiation. Results were compared to C57Bl/6J mice given 14 Gy-abdominal radiation. Effects of elemental liquid diet feeding from the day prior to radiation until day 7 post-radiation were assessed in both models. Body weight and a custom-developed health score was assessed every day until day 21 post-radiation. Intestine was assessed histologically.

**Results:**

At day 3 after segment radiation, complete loss of crypts occurred in the targeted segment, while adjacent and remaining intestine in segment-radiated mice, and entire intestine of sham controls, showed no detectable epithelial damage. Liquid diet feeding was required for survival of mice after segment radiation. Liquid diet significantly improved survival, body weight recovery and normalization of intestinal epithelium after abdominal radiation. Mice given segment radiation combined with liquid diet feeding showed minimal body weight loss, increased food intake and enhanced health score.

**Conclusions:**

The segment radiation method provides a useful model to study ISC/crypt loss and long-term crypt regeneration and epithelial repair, and may be valuable for future application to ISC transplantation or to genetic mutants that would not otherwise survive radiation doses that lead to complete crypt loss. Liquid diet is a simple intervention that improves survival and facilitates long-term studies of intestine in mice after high dose abdominal or segment radiation.

## Introduction

Increasing attention has been given to the impact of radiation on the gastrointestinal (GI) tract due to concerns about exposure to radiation after an accident or due to terrorist activity and a need for medical countermeasures [1], [2]. In addition, GI complications are limiting factors in dose and frequency of radiation therapy for abdominal cancers [2]–[4]. The GI epithelium is one of the most highly proliferative tissues in the body with constant renewal of the epithelial lining occurring over 3–10 days depending on the species and the region of the GI tract. In the small intestine, epithelial renewal is driven by intestinal stem cells (ISCs) that include actively cycling crypt-based columnar cells (CBCs) located at the base of the crypts and ‘quiescent’ ISCs which are thought to reside primarily at the +4 position from the base of the crypt [5]. ISCs yield more rapidly dividing progenitors which differentiate in all four intestinal epithelial cell (IEC) lineages, *i.e.* goblet cells, paneth cells, enteroendocrine cells and enterocytes, all subserving critical digestive, absorptive, barrier and defense functions [5]. Radiation induces DNA damage which, when radiation dose is sufficiently high, leads to death of a majority of cells within the crypt, and subsequent loss of villi and severe damage to intestinal epithelium. Improved understanding of mechanisms or strategies that promote crypt regeneration and epithelial repair after irradiation is highly desirable [1], [3].

Recent identification of ISC biomarkers [6]–[12] and development of ISC-reporter mouse models have led to major advances in the ISC field. However much is still to be learned about the specific role of ISCs during crypt regeneration following injury including the molecular pathways involved. Total Body Irradiation (TBI) and abdominal irradiation are considered gold standard methods to study ISC biology during crypt regeneration and subsequent epithelial repair in rodent models. High doses of TBI or abdominal radiation similarly induce massive or complete loss of small intestinal crypts followed by clonal expansion of single surviving ISCs which form regenerating epithelial microcolonies that repopulate the entire epithelium [13], [14]. Abdominal irradiation presents some advantages for studying the roles of ISCs during crypt regeneration and mucosal repair. Restricting radiation exposure to the abdomen attenuates bone marrow damage caused by radiation that has been suggested to sensitize mice toward lethality from gastrointestinal (GI) syndrome [15]. GI syndrome is characterized clinically by anorexia, vomiting, diarrhea, dehydration, systemic infection, and, in extreme cases, septic shock and death [2]. However, even abdominal irradiation at high doses (≥14 Gy), which are necessary to achieve complete crypt ablation in mice, can also lead to lethality due primarily to GI syndrome [14], [16]. The time of survival following such abdominal irradiation is strain-dependent [17] with death or need for euthanasia typically occurring between days 5 and 8 post-radiation in the commonly used C57BL/6 strain [18]. This short survival time limits long-term studies of regenerative processes since it permits only assessment of early phases of crypt and epithelial regeneration, which most commonly employs crypt microcolony assays, also called crypt survival assays [19]. A complete understanding of the mechanisms driving epithelial regeneration and repair, as well as the complications due to exposure of the intestine to high dose radiation would benefit from the ability to study later phases of regeneration involving intestinal epithelial hyperplasia and hyper-proliferation and ideally, times associated with complete normalization of the intestinal epithelial architecture. Furthermore the typically short time of survival after high dose TBI or abdominal radiation is also problematic to study the impact of genetic manipulations that may delay, impair or prevent crypt regeneration/mucosal repair or to study long-term consequences of radiation such as fibrosis.

In the present work, we developed and validated a method of radiation localized to an intestinal segment that induces complete crypt loss within the targeted segment but minimally impairs the health of wild type mice. We also compared chow feeding *versus* elemental liquid diet feeding in an abdominal radiation model and demonstrated that liquid diet significantly improves survival, body weight recovery and normalization of intestinal epithelium after high dose abdominal irradiation.

## Materials and Methods

### Animals

All experiments were performed on adult male or female C57BL/6J mice (6–10 week old) which were obtained from The Jackson Laboratory (Bar Harbor, ME). All animal experiments were carried out in accordance with the recommendations in the Guide for the Care and Use of Laboratory Animals of the National Institutes of Health. The protocol was approved by the University of North Carolina School of Medicine Institutional Animal Care and Use Committee (IACUC protocol number 10–197) and included criteria for euthanasia to minimize suffering.

### Intestinal Segment Radiation and Diet

A custom-designed lead shield with an opening allowing the isolation and subsequent radiation of a specific intestinal segment was purchased from Nuclead Co. Inc. and Sharp Manufacturing Inc. (West Bridgewater, MA) (Figure S1). Surgery was performed under sterile conditions using inhaled 2% isoflurane and oxygen for anesthesia. Through a midline incision the intestines were eviscerated and a 2–3 cm long segment of small intestine located approximately 15 cm proximal to the cecum was exteriorized and placed on the shield covered with saline-humidified gauze (Figure S1). A lead lid was placed on the opening to ensure that all other regions of the body were completely covered while leaving the intestinal segment exposed for radiation (Figure S1). The exteriorized segment was subjected to a single dose of 14 Gy radiation using an XRad 320 (Precision X-Ray, East Haven, CT) (Filter: 2 mm Al; 47 cm; 320 kV/s, 10 mA; 2.8 Gy/min). Sham controls had exteriorization of the segment and were placed into the lead shield but were given no radiation. All mice were hydrated with warm intraperitoneal zosyn-containing saline (0.2% w/v) and the abdomen was closed. Mice subjected to segment radiation were divided into normal chow fed and liquid diet fed groups. Normal chow was Prolab Isopro RMH 3000 (LabDiet, Henderson, CO) (see Table 1 for nutrient composition), while the elemental liquid diet was Nutren 1.0 (Vanilla, Nestlé Nutrition, Vevey, Switzerland), diluted in water 1∶2 (See Table 1 for nutrient composition). Liquid diet was substituted for chow on the day prior to radiation and mice were maintained on liquid diet for 7 days after radiation, and then returned to normal chow. Liquid diet was made up fresh and replaced every day. Also, the intake of liquid diet per mouse was measured every day. We note that due to high mortality of segment-radiated mice fed with normal chow only one experiment was performed in this model and histological analyses in mice given segment radiation focused on mice given liquid diet. Tissue was harvested for histology at 3 days after segment radiation, a time known to be associated with complete loss of crypts following 14 Gy radiation in high dose TBI or abdominal radiation models [11], to validate specific localization of the damaged zone and that radiation induced complete loss of crypts, and at 21 days after radiation, a time later than typically analyzed after high dose radiation, to study epithelial normalization.

**Table 1:** Nutrient composition of normal chow and elemental liquid diet.

	Composition			Daily intake*	
	Normal Chow	Liquid Diet		Normal Chow	Liquid Diet
	Prolab Isopro RMH 3000	Nutren 1.0		Prolab Isopro RMH 3000	Nutren 1.0
**Protein (%)**	22.5	4	**(g)**	1.125	0.72
**Carbohydrate (%)**	56	12.72	**(g)**	2.8	2.290
**Fat (%)**	11.8	3.8	**(g)**	0.59	0.684
**Sodium (%)**	0.26	0.088	**(mg)**	13	15.768
**Potassium (%)**	0.91	0.124	**(mg)**	45.5	22.32
**Vitamin A (IU/gm)**	29	3.2	**(IU)**	145	57.6
**Vitamin C (mg/gm)**	NA	0.14	**(mg)**	NA	2.52
**Calcium (%)**	1	0.067	**(mg)**	50	12.024
**Iron (ppm)**	380	12	**(mg)**	1.9	0.216
**Vitamin D (IU/gm)**	2.4	0.268	**(IU)**	12	4.824
**Vitamin E (IU/kg)**	75	28	**(IU)**	0.375	0.504
**Vitamin K (ppm)**	1.9	0.05	**(ug)**	9.5	0.9
**Thiamin (ppm)**	10	2	**(mg)**	0.05	0.036
**Riboflavin (ppm)**	14	2.4	**(mg)**	0.07	0.043
**Niacin (ppm)**	63	28	**(mg)**	0.315	0.504
**Vitamin B6 (ppm)**	7.6	4	**(mg)**	0.038	0.072
**Folic acid (ppm)**	1.2	0.54	**(ug)**	6	9.72
**Vitamin B12 (ug/kg)**	75	8	**(ug)**	0.375	0.144
**Biotin (ppm)**	0.38	0.4	**(ug)**	1.9	7.2
**Pantothenic acid (ppm)**	13	14	**(mg)**	0.065	0.252
**Phosphorus (%)**	1.19	0.067	**(mg)**	59.5	12.024
**Iodine (ppm)**	0.98	0.1	**(ug)**	4.9	1.8
**Magnesium (%)**	0.24	0.027	**(mg)**	12	4.824
**Zinc (ppm)**	120	14	**(mg)**	0.6	0.252
**Selenium (ppm)**	0.21	0.04	**(ug)**	1.05	0.72
**Copper (ppm)**	12	1.4	**(mg)**	0.06	0.025
**Manganese (ppm)**	96	2.72	**(mg)**	0.48	0.049
**Chromium (ppm)**	1.4	0.04	**(ug)**	7	0.72
**Molybdenum (ppm)**	NA	0.12	**(ug)**	NA	2.16
**Chloride (%)**	0.44	0.12	**(mg)**	22	21.6
**L-carnitine (ppm)**	NA	80	**(mg)**	NA	1.44
**Taurine (%)**	0.02	0.008	**(mg)**	1	1.44
**Choline (ppm)**	1600	452	**(mg)**	8	8.136
**Sulfur (%)**	0.26	NA	**(mg)**	13	NA
**Fluorine (ppm)**	16	NA	**(mg)**	0.08	NA
**Cobalt (ppm)**	0.27	NA	**(ug)**	1.35	NA
**Carotene (ppm)**	2.6	NA	**(ug)**	13	NA

* Daily intake was estimated for 5 grams of normal chow and 18 ml of liquid diet eaten/drunk per day.

### Abdominal Radiation and Diet

The segment radiation model was compared to abdominal radiation. The abdominal radiation model was also used to study the impact of liquid diet *versus* normal chow on overall health and long-term mucosal healing. Radiation experiments were performed under isoflurane anesthesia and anesthetized mice were placed in the radiator so that only the abdomen lay in the radiated zone as previously described [11]. Mice were given a single dose of 14 Gy irradiation using the XRad 320 (Precision X-Ray, East Haven, CT) (Filter: 2 mm Al; 47 cm; 320 kV/s, 10 mA; 2.8 Gy/min) as described above. Body weight was recorded at the start of the experiment and every day after radiation. Pilot studies indicated that mice fed normal chow typically lost up to 25% body weight until day 6 post-irradiation when body weight started to increase towards normal. Our approved IACUC protocol therefore permitted an exception to euthanize mice only if they lost more than 25% body weight. Mice subjected to abdominal radiation where divided into two groups. One was fed normal chow throughout the study and the other liquid diet from the day before radiation until 7 days post-radiation followed by a return to normal chow (See Table 1 for nutrient composition), as for the segment radiation studies described above. Mice were followed for 21 days after radiation to assess long-term health, and epithelial repair and normalization. A subset of animals was studied at day 4 post-radiation to assess survival of ISCs and formation of regenerating crypts by performing microcolony assays (see below).

### Health Score

To assess the health of the mice, four objective criteria were defined (Table 2). These included a score for activity (active = 1 and stationary = 0), posture (normal = 1 and hunched = 0), pelage (normal grooming, smooth and healthy-looking fur = 1 and lack of grooming and rough fur = 0) and dehydration (none = 1 and dehydrated = 0). Dehydration was tested by gently raising a small piece of dorsal skin and the mouse was considered dehydrated if the skin stayed up in a tent or only slowly retracted back to normal shape. The consistency and the objectivity of the health score evaluation was validated by 3 independent observers blinded to treatments. An overall health score ranging from 0 to 4 was determined every day during the 21 day-time course following segment or abdominal radiation. Any mouse assigned an overall health score of 0 was euthanized in accordance with our IACUC animal protocol. Survival data reflect those animals that did not meet the body weight or health score criteria for euthanasia.

**Table 2:** Health scoring criteria.

Criterion		Score
**ACTIVITY**	active	1
	stationary	0
**POSTURE**	normal	1
	hunched	0
**DEHYDRATION**	not dehydrated	1
	dehydrated	0
**PELAGE**	smooth	1
	rough	0

In order to evaluate the effects of radiation on health of mice, four objective criteria were used: activity (**active** or **stationary**), posture (**normal** or **hunched**), dehydration (**not dehydrated** or **dehydrated**) and pelage/hair coat (**smooth** or **rough**). Health scores were thus comprised between 4 (healthy mouse) and 0 (morbid mouse).

### Tissue Harvest for Histology

Mice were euthanized with a lethal dose of Nembutal (150 µg/g body weight). The entire small intestine was collected on ice, flushed with ice cold 1X phosphate buffered saline (PBS, 0.137 M NaCl, 3 mM KCl, 8 mM Na_2_HPO_4_, 2 mM KH_2_PO_4_, pH 7.4). The small intestine was divided into 3 segments corresponding to duodenum, jejunum and ileum. Tissues were opened longitudinally and fixed in in fresh 4% paraformaldehyde (PFA) in 1X PBS overnight at 4°C. Tissues were then rinsed in 1X PBS and cryoprotected by sequential incubations in 10% sucrose and 30% sucrose overnight at 4°C. Tissues were embedded in Optimal Cutting Temperature medium (OCT) as Swiss Rolls [20]. OCT-embedded tissues were then frozen on dry ice and stored at −80°C prior to cryosectioning. Sections (≈7 µm) were cut on a cryostat and placed on positively charged microscope slides. Sections were stained with hematoxylin and eosin (H&E) to visualize crypt and villus morphology or with Sirius red to assess collagen deposition and score the severity of fibrosis following established methods [21], [22]. Images were captured on Zeiss AXI0 microscope (Imager.A2, Zeiss, Oberkochen, Germany) fitted with ProgRes CF Scan digital camera (Jenoptik Laser Optik Systeme GmbH, Jena, Germany). The objective lens used was 5X (EC Plan Neofluar, Zeiss) with numerical apertures of 0.16.

### Microcolony Assays

To assess whether liquid diet impacted survival of ISCs and formation of regenerating crypts, a subset of abdominally-radiated mice given normal chow or liquid diet were euthanized at 4 days after irradiation, a time known to be associated with formation of regenerating microcolonies following 14 Gy radiation [11], [23]–[25]. Small intestine was processed for microcolony assays following established methods [19], [26]. Mice were injected with bromodeoxyuridine (BrdU) (200 mg/kg body weight) 90 minutes prior to euthanasia and the entire small intestine was collected on ice, flushed with ice cold 1X PBS and divided into 4 equal segments. The 3^rd^ segment from the proximal end (distal jejunum) was divided into 1 cm long cross-sections that were fixed in 10% Zinc-formalin overnight at 4°C. Cross sections were paraffin-embedded, sectioned and stained for BrdU (mouse monoclonal anti-BrdU; 1∶100; Invitrogen) with diaminobenzidine (DAB; Thermo Scientific) using an ABC Elite reaction kit (Vector). Sections were analyzed using a Zeiss AXI0 microscope (Imager.A2, Zeiss, Oberkochen, Germany). A crypt was considered to be a viable surviving crypt if it contained five or more BrdU-positive cells [19], [26]. The number of surviving crypts was determined in each cross section and the mean number of surviving crypts per cross section was assessed for each animal.

#### Statistics

Data were expressed as the mean ± SEM. Mann-Whitney test, paired t-test, one way ANOVA or two-way ANOVA were performed to compare different groups as indicated in the results or figure legends. A p-value of less than 0.05 was considered statistically significant.

## Results

### The Segment Radiation method Allows Specific and Complete Ablation of Crypts in the Targeted Intestinal Segment

**Figure 1:**
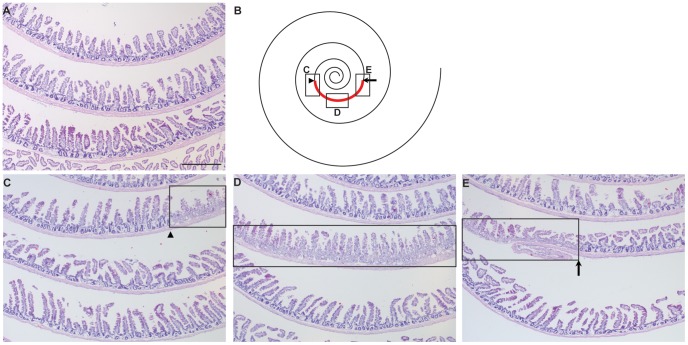
The segment radiation method allows specific and total crypt ablation in a pre-determined intestinal segment. A. Illustrative photograph of H&E-stained Swiss-rolled jejunum from sham controls shows that no damage was induced by the segment exteriorization procedure (n = 3). Scale Bar: 500 µm. B. The schematic represents Swiss-rolled jejunum (**black spiral**). The radiated segment is shown in **red** between the **arrow head** and the **arrow**. The 3 **rectangles** (**C**, **D** and **E**) illustrate the localization of the radiated segment in the photographs shown in C, D and E. Illustrative photographs of H&E-stained Swiss-rolled jejunum collected from a mouse at day 3 post-radiation demonstrates that the segment radiation procedure induced specific and complete ablation of crypts in the targeted portion of the jejunum delimited by the arrowhead and the arrow, while the rest of the tissue showed normal epithelial architecture (n = 3). In these experiments, all mice (segment-radiated or sham controls) received liquid diet.

We first sought to assess whether the segment radiation method led to damage specifically in the radiated segment. Three independent mice were subjected to segment radiation and compared with sham controls. Intestinal tissues were collected at day 3 post-radiation, time associated with complete crypt ablation in jejunum after 14 Gy abdominal radiation [11]. H&E sections of Swiss-rolled intestines revealed no damage in sham controls (Figure 1A and Figure S2), demonstrating that the exteriorization procedure does not affect the mucosa. However, in segment radiated mice, total loss of crypts was observed exclusively within the targeted segment, while the rest of the intestine was unaffected (Figure 1B−E and Figure S2).

### The Segment Radiation Method and Liquid Diet Feeding Permit Long-term Post-Radiation Studies

To validate that the segment radiation method allows long-term post-radiation studies, we then monitored mice for 21 days after 14 Gy abdominal or segment radiation. Mice were fed either with normal chow for all 21 days of the study or with elemental liquid diet from the day prior to radiation and the 7 consecutive days followed by return to normal chow for the rest of the study.

#### Liquid diet leads to maximal survival after both segment and abdominal radiation

**Figure 2:**
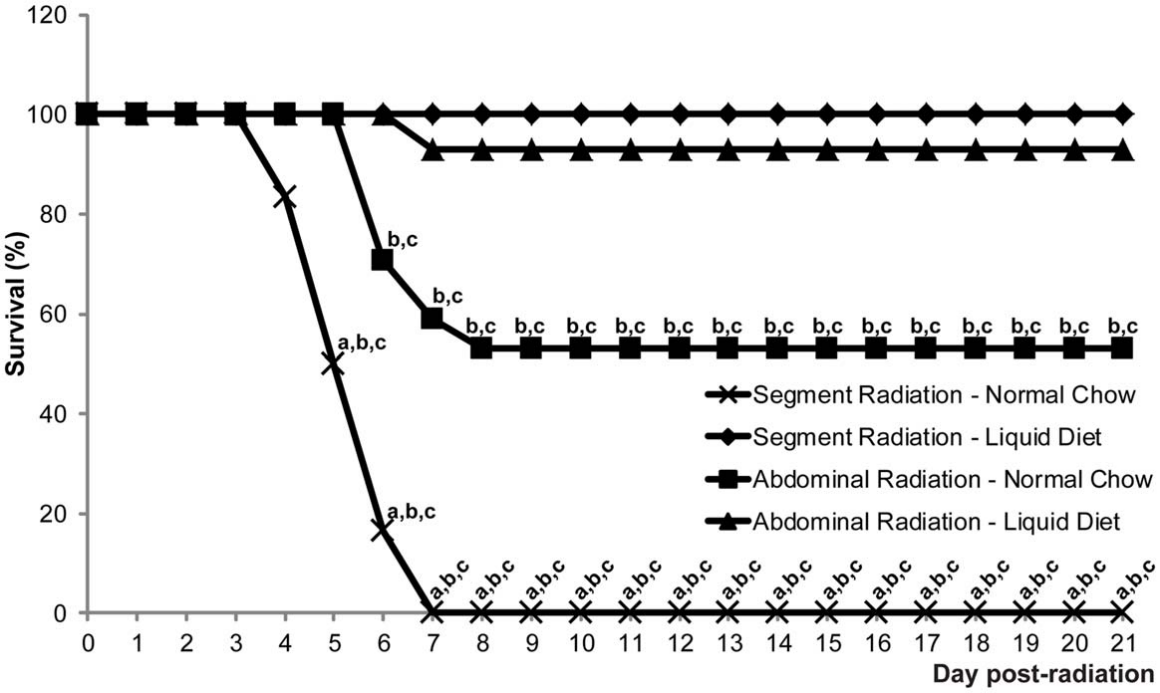
Liquid diet leads to maximal survival after both segment and abdominal radiation. Graphs show percent survival at different times after abdominal or segment radiation in mice given normal chow or liquid diet (from the day prior to radiation until day 7 post-radiation). 52.9% of the mice fed with normal chow and abdominally irradiated survived for 21 days post-radiation while none survived when fed with normal chow and locally radiated on a determined intestinal segment. Liquid diet treatment significantly improved survival after both local and abdominal radiation (n≥6: Abdominal Radiation-Normal Chow n = 17; Abdominal Radiation-Liquid Diet n = 14; Segment Radiation-Normal Chow n = 6; Segment Radiation-Liquid Diet n = 14; Mann-Whitney; **a**: p<0.05 *vs.* Abdominal Radiation-Normal Chow; **b**: p<0.05 *vs.* Abdominal Radiation-Liquid Diet; **c**: p<0.05 *vs.* Segment Radiation-Liquid Diet).

Survival was calculated as the percentage of mice that did not require euthanasia due a loss of body weight greater than 25% or an overall health score of 0. After segment radiation, 100% mice survived when they were placed on liquid diet, while none survived beyond day 7 when fed with normal chow (Figure 2), which was likely due to ileus as a result of bowel manipulation, leading to impacted chow and obstruction. Note that because of the high mortality in segment-radiated mice fed with normal chow we did not pursue additional studies in these mice or in additional groups of mice given normal chow after segment radiation. After abdominal radiation, only 52.9% of mice fed with normal chow survived (Figure 2). Liquid diet drastically and significantly improved survival since 92.9% of mice that were abdominally radiated and fed with liquid diet survived (Figure 2).

#### Segment radiation combined with liquid diet feeding leads to minimal body weight loss after radiation

**Figure 3:**
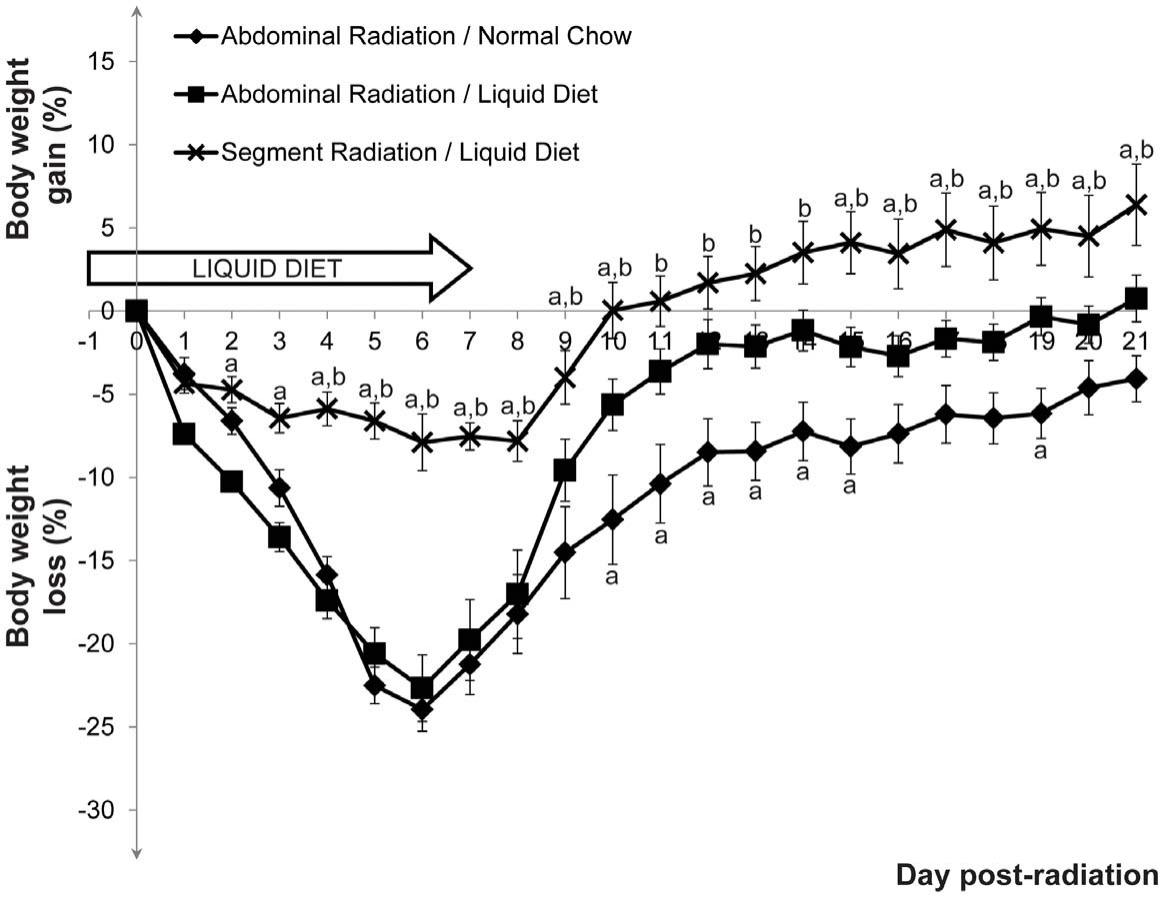
The segment radiation method combined with liquid diet induces minimal body weight loss. Graphs show percent body weight loss (mean ± SEM) at different times after irradiation in abdominally-radiated mice given normal chow or liquid diet and in segment-radiated mice given liquid diet. Mice locally radiated on an intestinal segment and fed with liquid diet exhibited minimal body weight loss and rapid body weight recovery after radiation compared with mice abdominally radiated. Liquid diet significantly improved body weight recovery of mice treated with abdominal radiation (n≥9: Abdominal Radiation-Normal Chow n = 12; Abdominal Radiation-Liquid Diet n = 9; Segment Radiation-Liquid Diet n = 9; Two Way ANOVA; **a**: p<0.05 *vs.* Abdominal Radiation-Liquid Diet; **b**: p<0.05 *vs.* Abdominal Radiation-Normal Chow).

In all groups, radiation induced body weight loss which peaked at day 6 post-radiation. Between days 2 and 6 post-radiation, mice radiated on a targeted segment and fed with liquid diet exhibited minimal body weight loss when compared to abdominally-radiated mice fed with either normal chow or liquid diet (7.9% ±1.7 (segment radiation) vs. 24.0% ±1.3 (abdominal radiation- normal chow) and 22.7% ±2.0 (abdominal radiation- liquid diet) body weight loss at day 6 post-radiation) (Figure 3). Mice radiated on a targeted segment consistently began to regain body weight at day 9 post-radiation. Interestingly, they continued gaining weight until the end of the study and at 21 days post-radiation, they showed increased body weight when compared to day 0. Comparisons between abdominally-irradiated mice given either liquid diet or normal chow revealed similar body weight loss over 9 days after radiation, but thereafter the mice on liquid diet more rapidly gained weight (Figure 3).

#### Segment radiation combined with liquid diet feeding improves health score of mice after radiation

**Figure 4:**
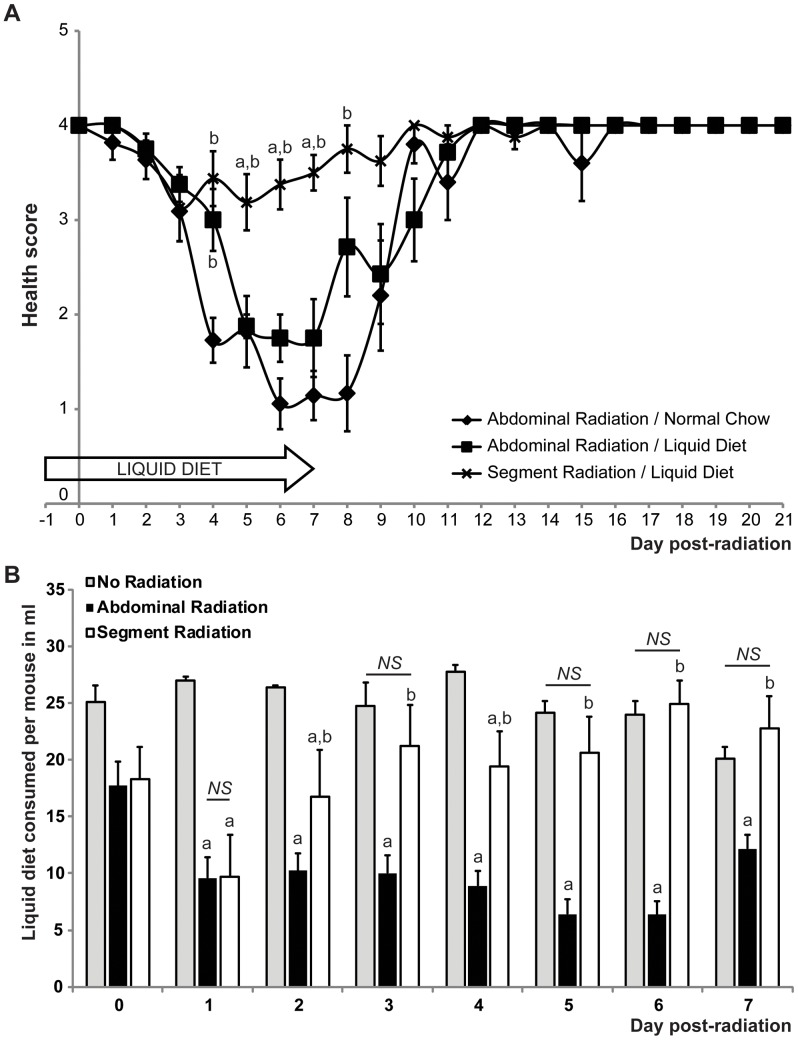
The segment radiation method combined with liquid diet improves health post-radiation. A. Graphs show health scores (mean ± SEM) of abdominally-radiated mice given normal chow or liquid diet and segment-radiated mice given liquid diet at different times after irradiation. Mice locally radiated on a targeted intestinal segment and fed with liquid diet exhibited significantly improved health score between day 4 and day 8 post-radiation as compared with abdominally radiated mice. Liquid diet tends to slightly improve health score between day 4 and day 8 post abdominal radiation (n≥8: Abdominal Radiation-Normal Chow n = 11; Abdominal Radiation-Liquid Diet n = 8; Segment Radiation-Liquid Diet n = 8; ANOVA; **a**: p<0.05 *vs.* Abdominal Radiation-Liquid Diet; **b**: p<0.05 *vs.* Abdominal Radiation-Normal Chow). B. Bar Graphs show amounts of liquid diet consumed per mouse per day in ml (mean ± SEM) in non-irradiated mice or mice given abdominal or segment radiation. Mice locally radiated on an intestinal segment exhibited significantly increased intake of liquid diet as compared with abdominally-radiated mice and had similar intake to non-irradiated control mice by 3–5 days after irradiation (n≥12: No Radiation n = 13; Abdominal Radiation n = 14; Segment Radiation n = 12; Mann-Whitney; **a**: p<0.05 *vs.* No Radiation; **b:** p<0.05 *vs.* Abdominal Radiation; ***NS***: not significant).

To assess the health of mice after radiation, we evaluated activity, posture, dehydration and pelage of the mice (Table 2). Radiation induced a decrease in health score in all groups (Figure 4A). Mice locally radiated on a targeted segment exhibited a minimum decline in health score from the maximal health score of 4 such that the lowest mean health score was 3.13±0.35 at day 3 post-radiation and then rapidly returned to normal (Figure 4A). Abdominally-radiated mice fed with either normal chow or liquid diet showed significantly lower health scores compared with segment-radiated mice with minimum health scores of 1.06±0.27 (normal chow) and 1.75±0.25 (liquid diet), respectively, at day 6 post radiation. Liquid diet tended to improve health score between days 4 and 8 after abdominal radiation (Figure 4A). By day 12 post-radiation, all groups were back to maximal health score. Food intake can also be considered as a sign of health and we compared the amount of liquid diet consumed per mouse per day between non-irradiated, abdominally- or segment-radiated mice. Both segment- and abdominally-radiated mice exhibited decreased intake of liquid diet during the first two days post-radiation as compared to non-irradiated controls. In contrast to abdominally-radiated mice, segment-radiated mice consumed volumes of liquid diet similar to non-irradiated controls as early as day 3 post-radiation. Also they exhibited significantly greater intake of liquid diet than abdominally-radiated mice from day 2 to day 7 post-radiation, which was the last day on liquid diet (Figure 4B). This is likely a key contributor to the improved body weight of segment-radiated mice noted in Figure 3.

#### Liquid diet promotes normalization of the intestinal epithelial architecture following abdominal radiation

**Figure 5:**
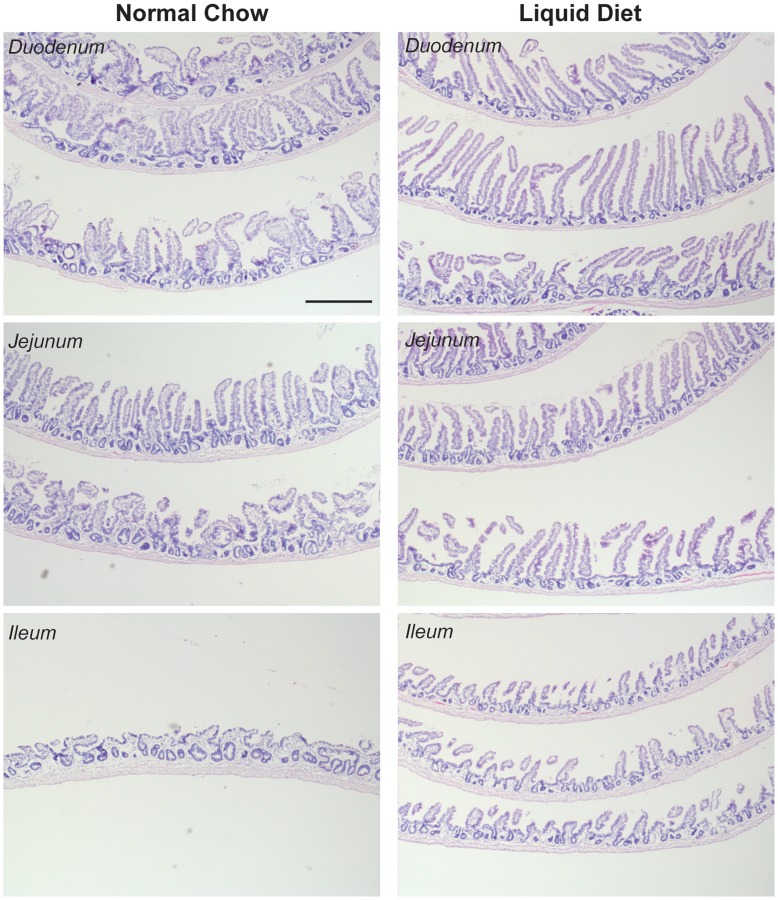
Liquid diet promotes normalization of the intestinal epithelium after abdominal radiation. H&E staining of Swiss-rolled intestines at day 21 post-abdominal radiation showed enlarged crypts as well as blunted villi in mice fed with normal chow and overall more abnormal mucosal epithelium when compared with mice fed with liquid diet (n = 3). Scale Bar: 500 µm.

Intestines of abdominally-radiated mice given normal chow or liquid diet (from the day prior to radiation until day 7 post-radiation) were studied at day 21 to assess mucosal healing and epithelial normalization. Mice given liquid diet showed normal crypt-villus architecture throughout duodenum, jejunum and ileum (Figure 5). In contrast mice fed with normal chow exhibited abnormal crypt villus architecture in duodenum, jejunum and ileum (Figure 5). Crypts were enlarged and villi were blunted, and this was particularly marked in the ileum (Figure 5). These data suggest that liquid diet promotes normalization of the epithelium after high dose abdominal radiation. To assess whether this was due to a differential initial impact of abdominal radiation between mice on liquid diet *vs*. normal chow, we compared complete blood count and microcolony formation, *i.e.* crypt survival, at day 4 post-radiation. No significant difference was observed in complete blood count between the two groups, both exhibiting white blood cell count below normal range, demonstrating that abdominal radiation resulted in similarly impaired immune function in liquid diet- and normal chow-fed groups (Table 3). Also microcolony assays revealed numbers of regenerating microcolonies per crypt section that did not differ significantly between abdominally-radiated mice fed with either normal chow or liquid diet, indicating similar crypt survival at day 4 post-radiation (25.0±6.5 (normal chow) *vs.* 22.8±8.8 (liquid diet) regenerating crypts per cross section; n = 4; Mann-Whitney; not significant). Together, these data strongly suggest that liquid diet treatment promoted normalization of the intestinal epithelium during later phases of mucosal healing after high dose abdominal radiation, while it did not affect initial clonogenic crypt survival occurring at earliest phases.

**Table 3:** Liquid diet does not impact radiation-induced changes in complete blood count.

		Normal Chow			Liquid Diet			
	Normal Ranges	mean	+/−	SEM	mean	+/−	SEM	p-value
**White Blood Cell count (10^3^/uL)**	2.6∶ 10.1	0.63	+/−	0.17	0.95	+/−	0.43	0.51
**Lymphocytes (10^3^/uL)**	1.3∶ 8.4	0.30	+/−	0.11	0.53	+/−	0.33	0.59
**Granulocytes (10^3^/uL)**	0.4∶ 2.0	0.30	+/−	0.09	0.50	+/−	0.10	0.20
**Monocytes (10^3^/uL)**	0.0∶ 0.3	0.13	+/−	0.04	0.13	+/−	0.03	1.00
**Lymphocytes (%)**	0.0∶ 99.9	38.67	+/−	11.23	37.47	+/−	9.56	0.93
**Granulocytes (%)**	0.0∶ 99.9	49.40	+/−	13.29	53.97	+/−	8.89	0.73
**Monocytes (%)**	0.0∶ 99.9	11.93	+/−	3.13	8.57	+/−	2.28	0.32
**Hematocrit (%)**	32.8∶ 48.0	30.58	+/−	2.96	34.78	+/−	1.80	0.27
**Mean Cell Volume (fl)**	42.3∶ 55.9	40.55	+/−	0.32	40.28	+/−	0.18	0.48
**Red Blood Cells (10^6^/uL)**	6.5∶ 10.1	7.53	+/−	0.69	8.63	+/−	0.46	0.24
**Hemoglobin (g/dL)**	10.1∶ 16.1	12.23	+/−	1.00	13.60	+/−	0.61	0.29
**Mean Corpuscular Volume (pg)**	13.7∶ 18.1	16.25	+/−	0.18	15.80	+/−	0.20	0.15
**Mean Corpuscular Hemoglobin Concentration (g/dL)**	29.5∶ 35.1	40.18	+/−	0.61	39.20	+/−	0.35	0.22
**Red Blood Cell Distribution Width (%)**	0.0∶ 99.9	19.28	+/−	0.41	19.33	+/−	0.24	0.92
**Mean Platelet Volume (fl)**	0.0∶ 99.9	6.10	+/−	0.07	5.98	+/−	0.02	0.15
**Platelet (10^3^/uL)**	780∶ 1540	645.75	+/−	109.03	636.50	+/−	28.90	0.94

Complete blood count performed at day 4 following abdominal radiation showed no difference between mice fed with normal chow or liquid diet (Abdominal Radiation-Normal Chow n = 4; Abdominal Radiation-Liquid Diet n = 4; t-test).

#### Locally-radiated intestinal segment exhibits mild fibrosis at 21 days post-radiation

**Figure 6:**
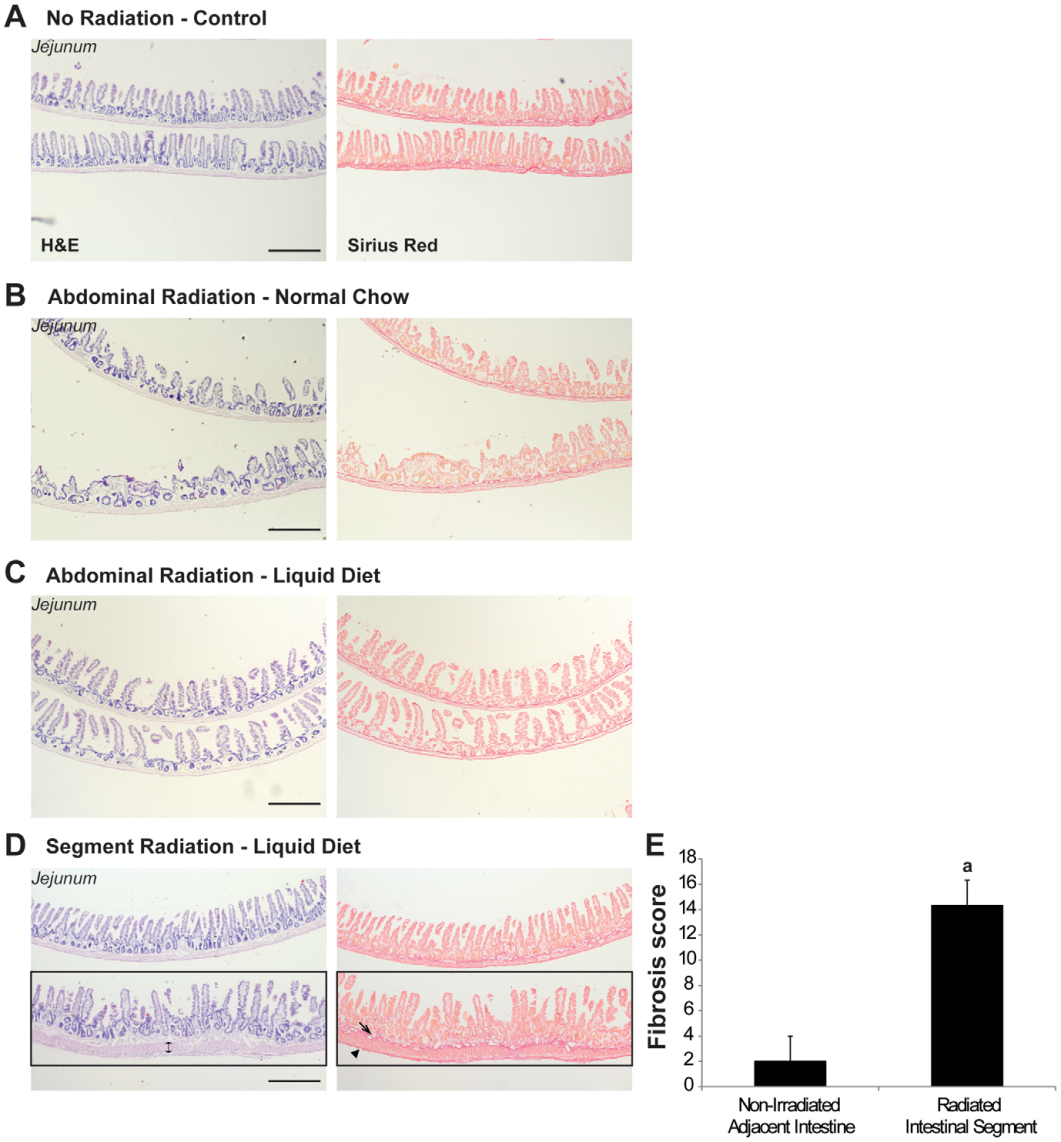
Locally-radiated intestinal segments exhibit mild fibrosis at day 21 post-radiation. A–C. H&E staining (*left panels)* and Sirius red staining (*right panels*) revealed no evident sign of fibrosis in distal jejunum of abdominally-radiated mice fed with normal chow (**B**) or liquid diet (**C**) at day 21 post-radiation as compared to non-irradiated controls (**A**). Scale Bar: 500 µm. D. *Left panel.* However, in mice given segment radiation, the radiated segment (**black rectangle**) was still distinguishable from adjacent non-irradiated intestine at day 21 post-radiation by H&E staining and exhibited a thickening of the muscular wall (**double headed arrow)**. *Right panel.* Sirius red staining revealed an increase in collagen deposition within the submucosa (**arrow**) and the serosa (**arrowhead**) of the radiated segment (**black rectangle**). Scale Bar: 500 µm. E. Quantification of the extent of the collagen deposition demonstrated a significant increase in fibrosis in the radiated segment *vs.* non-irradiated adjacent tissue at 21 days after irradiation (n = 3; paired t-test; **a**: p<0.05).

In segment-radiated mice, the radiated intestinal segment was still identifiable by H&E staining at day 21 post-radiation and did not exhibit complete normalization of architecture of the mucosa and bowel wall as compared with non-irradiated tissue adjacent to the radiated segment (Figure 6D). Sirius red staining revealed a consistent increase in submucosal and serosal collagen deposition, as well as a thickening of the muscular layer, also visible by H&E, in the radiated segment as compared to adjacent tissue (Figure 6D). Fibrosis scoring [21], [22] quantitatively demonstrated that there was a significant increase in collagen deposition in the radiated segment at 21 days post-radiation compared with non-irradiated adjacent tissue (Figure 6E). Interestingly, distal jejunum of abdominally-radiated mice fed with normal chow or liquid diet showed no evident sign of fibrosis as compared to non-irradiated controls (Figure 6A−C).

## Discussion

Recent identification of ISC biomarkers offers new opportunities to investigate the roles of ISCs and functionally relevant pathways during crypt regeneration and mucosal repair after radiation. Conventional *in vivo* radiation models induce a high mortality early during the days following high dose radiation and therefore do not allow the study of intermediate and late phases of mucosal repair. The results presented in this study demonstrate that elemental liquid diet feeding markedly improved survival after high dose abdominal radiation. Importantly, we report a novel model of radiation of a specific small intestinal segment that induces localized complete crypt ablation with minimal adverse effects on the health of mice, thus permitting long-term studies of ISCs, crypt regeneration and mucosal healing after radiation-induced crypt ablation in the small intestinal epithelium.

There is very limited information about the impact of nutrition and hydration on survival or mucosal healing after irradiation. However, liquid diet composed of equal volumes of evaporated milk and water with additional glucose has been shown to slightly improve survival of mice after lethal irradiation of the head [27]. Also germ-free mice, but not conventional mice, showed significantly decreased mortality and improved body weight loss after TBI when supplemented from 5-weeks after birth with a liquid diet containing all known essential nutrients required by mice, in addition to normal chow, and maintained on this supplemental diet until the end of the experiment [17]. This is consistent with our findings that liquid diet feeding significantly promoted survival and accelerated normalization of body weight after abdominal radiation, although in our study mice were fed solely with liquid diet from the day prior to radiation until 7 days post-radiation, followed by a return to normal chow until the end of the study at 21 days post-abdominal radiation. Interestingly the microcolony assay results indicate that liquid diet did not impact crypt survival and regenerating microcolony formation during the earliest phases of crypt regeneration after abdominal radiation. Thus the beneficial effects of liquid diet appear not to originate from an impact on early regenerating ISC/crypts. However, our results in the abdominal radiation model demonstrate that liquid diet feeding promoted histological normalization of the intestinal epithelial architecture at day 21 after abdominal radiation when compared with littermates fed normal chow. Further studies will be required to define the mechanisms underlying the beneficial effect of liquid diet on epithelial normalization. Since liquid diet had no detectable impact on early phases of crypt regeneration and since mice were back on normal chow at day 7 after abdominal radiation, it seems possible that these mechanisms may involve protective effects of liquid diet on ISC/intestinal epithelial cells at times later than 4 days post-radiation assessed here, or effects on cell types other than ISCs or intestinal epithelial cells, such as resident immune cells or other cell types present in the intestinal epithelial cell microenvironment. We did not investigate specific effects of particular components of the liquid diet since it was shown in Walburg et al. 1966 [17] that none of the major groups of essential nutrients contained in a liquid diet was capable alone of modifying the mortality pattern after TBI radiation. One difference in the nutrient composition between liquid diet and normal chow used in the present study is that liquid diet contains vitamin C, while vitamin C is undetectable in chow diet. A recent study reported that a 3 day or 12 hour pre-treatment with vitamin C markedly improved survival of mice after 14 Gy TBI [28]. Estimated daily intake of vitamin C in the liquid diet fed animals in the present study approximates the vitamin C supplementation in the study of Yamamoto et al. 2010 [28]. However in that study, vitamin C pre-treatment only had a beneficial effect if mice were subjected to BM transplantation [28]. Therefore, while we cannot exclude a role of vitamin C in the beneficial effects of liquid diet feeding observed in our study, it seems unlikely that these beneficial effects are solely due to vitamin C since mice in the current study did not require BM transplantation.

A recent report issued following a workshop of the Centers for Medical Coutermeasures against Radiation (CMCRs) stressed the importance of the animal models used for the identification and the assessment of the effects of pharmacological agents on radiation-induced intestinal damage and subsequent repair [1]. In murine models, survival time after radiation has been shown to be strain-dependent with influence of both the genetic background and the resident microbiota [17], [29]–[32]. Also comparative studies become even more complicated when the field of radiation is considered, *i.e.* TBI *vs.* abdominal radiation. Indeed traditional views in the field consider that morbidity in abdominally-radiated animals is typically due to GI failure [14]. However, more recent findings suggest that abdominal radiation can also result in BM failure-induced death [33]. In TBI murine models, timing of morbidity is usually used as sole indicator distinguishing GI failure from BM failure [14]. Therefore, a recent report recommends that the dose-dependent mode of animal death after TBI or abdominal radiation should be constructed for each strain and for each experimental system based on autopsy examination [16]. The segment radiation model reported in the present study has the advantage that it minimally affects the health of the mice and permits specific evaluation of the impact of high dose radiation on the intestine. Other groups have used segment radiation in mice [34], [35]. However, to our knowledge, these studies did not combine the segment radiation procedure with a liquid diet treatment. As a consequence, these models only allowed short-term studies [34] or presented a high rate of mortality [35]. This is consistent with our findings that no mice survived the segment radiation method when fed normal chow. Experience with surgical models such as proximal bowel resection or ileo-cecal resection has been that post-surgical ileus leads to impaction of solid chow. In these models, liquid diet feeding before and after surgery dramatically improved survival [36]. Since radiation is known to alter gut motility [37], [38], and the segment radiation method involves bowel handling and radiation, we believe that ileus was the likely cause of death/need for euthanasia in segment-radiated mice fed with normal chow. We did not study tissue histology in these animals due to the limited number studied under this protocol. We cannot exclude that fibrosis contributed to high mortality since adhesions were noted in segment-radiated mice fed with normal chow at autopsy.

The present study is the first comprehensive documentation of the segment radiation technique combined with liquid diet in a mouse model that allows long-term studies of intestinal responses to high dose radiation. It is also the first demonstration of the value of a liquid diet treatment in both the abdominal and segment radiation procedures. Under the conditions we described, the segment radiation/liquid diet protocol allows analyses of ISC-mediated crypt regeneration in a context of healthy surrounding organs, including a grossly healthy, adjacent GI tract. More importantly, the segment radiation method allows analyses of non-irradiated and irradiated intestine of the same mouse. This provides a useful model for direct comparisons of crypt homeostasis and ISCs in uninjured *vs.* radiated and injured intestine of the same animal, with both regions of the intestine subjected to the same systemic environment. Also, the segment radiation model should prove valuable for assessing impact of nutritional or pharmacologic interventions on both the intestinal segment injured by radiation and the normal intestine distant from the site of radiation exposure. This has significant advantages for defining interventions that may specifically promote healing of radiated intestine. Furthermore the segment radiation model should prove useful to study impact of genetic modifications on responses of the intestine to radiation in mutant mice where phenotypic effects of the mutation on body size and growth, immune system or other aspects of physiology might preclude their ability to survive high dose TBI or abdominal radiation.

Intestinal fibrosis is a long-term potential complication of TBI or abdominal radiation exposure that can lead to bowel dysfunction and potentially bowel obstruction or intestinal failure. Analyses of intestinal fibrosis after high dose TBI or abdominal radiation in rodent models can be problematic due to the high morbidity in the early period after high dose radiation or due to obstruction or motility disorders in fibrotic intestine. Interestingly, in the segment radiation model, at 21 days post-radiation, we observed increased collagen deposition within the submucosa and serosa of the irradiated intestinal segment concomitant with a thickening of the muscularis, both features of fibrosis. This localized fibrosis within the specific radiated intestinal segment could prove very valuable for studying the mechanisms of radiation-induced fibrosis or testing anti-fibrotic therapies. It is perhaps surprising that the abdominally-radiated mice did not exhibit fibrosis at 21 days post-radiation. However this may reflect the time point selected and more studies and time points are needed to compare fibrosis in the abdominal and segment radiation models.

In summary, this work demonstrates that liquid diet feeding represents a simple and low cost intervention which promotes survival and allows long-term studies of intestinal epithelial repair in murine models after high dose abdominal radiation. Importantly the high dose segment radiation model induces complete localized ISC/crypt loss while minimally affecting the health of the mice, and may be extremely useful for the study of genetically engineered mice with defects in mucosal repair or physiological impairments that may otherwise preclude survival after high doses TBI or abdominal irradiation, as well as for testing therapeutic strategies to activate ISCs and promote crypt regeneration. This radiation model could also be of value for future application to ISC transplantation.

## Supporting Information

Figure S1 **Custom-made lead shield.** The lead shield was custom-designed so that it completely protects the mice from a 14 Gy radiation dose at a distance of 47 cm from the X-Ray source (320 kV/s, 10 mA; 2.8 Gy/min) equipped with a 2 mm Al filter. A. The opening in the center of the shield allows isolation and subsequent radiation of an exteriorized intestinal segment. B. The lead lid ensures complete cover of the mouse body during radiation of the exteriorized intestinal segment. C. The schematic illustrates the positioning of the mouse under the lead shield, the exteriorized segment on a humidified gauze and the lead lid covering the opening of the shield.
(TIF)

Figure S2 **Duodenum and ileum of sham controls and segment-radiated mice show no epithelial damage.** Illustrative photographs of H&E-stained Swiss-Rolled sections of duodenum and ileum of sham controls (A and B, respectively) demonstrate that the surgical procedure of the segment radiation method induces no damage to the intestinal epithelium (n = 3). Scale Bar: 500 µm. Similarly the duodenum (C) and the ileum (D) of mice radiated on a specific segment exhibited no epithelial damage, further attesting to the specificity of the segment radiation method.
(TIF)

## References

[1] Williams JP, Brown SL, Georges GE, Hauer-Jensen M, Hill RP, et al (2010) Animal models for medical countermeasures to radiation exposure. Radiat Res 173: 557–578. 20334528 10.1667/RR1880.1 PMC3021126

[2] Somosy Z, Horvath G, Telbisz A, Rez G, Palfia Z (2002) Morphological aspects of ionizing radiation response of small intestine. Micron 33: 167–178. 11567886 10.1016/s0968-4328(01)00013-0

[3] Theis VS, Sripadam R, Ramani V, Lal S (2010) Chronic radiation enteritis. Clin Oncol (R Coll Radiol) 22: 70–83. 19897345 10.1016/j.clon.2009.10.003

[4] Zimmerer T, Bocker U, Wenz F, Singer MV (2008) Medical prevention and treatment of acute and chronic radiation induced enteritis–is there any proven therapy? a short review. Z Gastroenterol 46: 441–448. 18461520 10.1055/s-2008-1027150

[5] van der Flier LG, Clevers H (2009) Stem cells, self-renewal, and differentiation in the intestinal epithelium. Annu Rev Physiol 71: 241–260. 18808327 10.1146/annurev.physiol.010908.163145

[6] Barker N, van Es JH, Kuipers J, Kujala P, van den Born M, et al (2007) Identification of stem cells in small intestine and colon by marker gene Lgr5. Nature 449: 1003–1007. 17934449 10.1038/nature06196

[7] Gracz AD, Ramalingam S, Magness ST (2010) Sox9 expression marks a subset of CD24-expressing small intestine epithelial stem cells that form organoids in vitro. Am J Physiol Gastrointest Liver Physiol 298: G590–600. 20185687 10.1152/ajpgi.00470.2009 PMC2867430

[8] Powell AE, Wang Y, Li Y, Poulin EJ, Means AL, et al (2012) The pan-ErbB negative regulator Lrig1 is an intestinal stem cell marker that functions as a tumor suppressor. Cell 149: 146–158. 22464327 10.1016/j.cell.2012.02.042 PMC3563328

[9] Sangiorgi E, Capecchi MR (2008) Bmi1 is expressed in vivo in intestinal stem cells. Nat Genet 40: 915–920. 18536716 10.1038/ng.165 PMC2906135

[10] Takeda N, Jain R, LeBoeuf MR, Wang Q, Lu MM, et al (2011) Interconversion between intestinal stem cell populations in distinct niches. Science 334: 1420–1424. 22075725 10.1126/science.1213214 PMC3705713

[11] Van Landeghem L, Santoro MA, Krebs AE, Mah AT, Dehmer JJ, et al (2012) Activation of two distinct Sox9-EGFP-expressing intestinal stem cell populations during crypt regeneration after irradiation. Am J Physiol Gastrointest Liver Physiol 302: G1111–1132. 22361729 10.1152/ajpgi.00519.2011 PMC3362093

[12] von Furstenberg RJ, Gulati AS, Baxi A, Doherty JM, Stappenbeck TS, et al (2011) Sorting mouse jejunal epithelial cells with CD24 yields a population with characteristics of intestinal stem cells. Am J Physiol Gastrointest Liver Physiol 300: G409–417. 21183658 10.1152/ajpgi.00453.2010 PMC3064119

[13] Hendry JH, Roberts SA, Potten CS (1992) The clonogen content of murine intestinal crypts: dependence on radiation dose used in its determination. Radiat Res 132: 115–119. 1410267

[14] Mason KA, Withers HR, McBride WH, Davis CA, Smathers JB (1989) Comparison of the gastrointestinal syndrome after total-body or total-abdominal irradiation. Radiat Res 117: 480–488. 2648450

[15] Terry NH, Travis EL (1989) The influence of bone marrow depletion on intestinal radiation damage. Int J Radiat Oncol Biol Phys 17: 569–573. 2528526 10.1016/0360-3016(89)90108-9

[16] Rotolo JA, Kolesnick R, Fuks Z (2009) Timing of lethality from gastrointestinal syndrome in mice revisited. Int J Radiat Oncol Biol Phys 73: 6–8. 19100919 10.1016/j.ijrobp.2008.09.009

[17] Walburg HE Jr, Mynatt EI, Robie DM (1966) The effect of strain and diet on the thirty-day mortality of x-irradiated germfree mice. Radiat Res 27: 616–629. 5934823

[18] Austin MK, Miller M, Quastler H (1956) Five- to eight-day radiation death in mice. Radiat Res 5: 303–307. 13359683

[19] Withers HR, Elkind MM (1970) Microcolony survival assay for cells of mouse intestinal mucosa exposed to radiation. Int J Radiat Biol Relat Stud Phys Chem Med 17: 261–267. 4912514 10.1080/09553007014550291

[20] Moolenbeek C, Ruitenberg EJ (1981) The “Swiss roll”: a simple technique for histological studies of the rodent intestine. Lab Anim 15: 57–59. 7022018 10.1258/002367781780958577

[21] Rigby RJ, Hunt MR, Scull BP, Simmons JG, Speck KE, et al (2009) A new animal model of postsurgical bowel inflammation and fibrosis: the effect of commensal microflora. Gut 58: 1104–1112. 19398439 10.1136/gut.2008.157636 PMC2752281

[22] Theiss AL, Fuller CR, Simmons JG, Liu B, Sartor RB, et al (2005) Growth hormone reduces the severity of fibrosis associated with chronic intestinal inflammation. Gastroenterology 129: 204–219. 16012948 10.1053/j.gastro.2005.05.019

[23] Bhanja P, Saha S, Kabarriti R, Liu L, Roy-Chowdhury N, et al (2009) Protective role of R-spondin1, an intestinal stem cell growth factor, against radiation-induced gastrointestinal syndrome in mice. PLoS One 4: e8014. 19956666 10.1371/journal.pone.0008014 PMC2777375

[24] Booth D, Potten CS (2001) Protection against mucosal injury by growth factors and cytokines. J Natl Cancer Inst Monogr: 16–20. 10.1093/oxfordjournals.jncimonographs.a003433 11694560

[25] Martin K, Potten CS, Roberts SA, Kirkwood TB (1998) Altered stem cell regeneration in irradiated intestinal crypts of senescent mice. J Cell Sci 111 (Pt 16): 2297–2303. 10.1242/jcs.111.16.2297 9683625

[26] Tustison KS, Yu J, Cohn SM (2001) Assessment of intestinal stem cell survival using the microcolony formation assay. Methods Mol Med 50: 267–273. 21318835 10.1385/1-59259-084-5:267

[27] Goepp RA, Fitch F (1963) Prevention of Death in Mice after Lethal Irradiation of the Head. Radiat Res 19: 670–675. 14065584

[28] Yamamoto T, Kinoshita M, Shinomiya N, Hiroi S, Sugasawa H, et al (2010) Pretreatment with ascorbic acid prevents lethal gastrointestinal syndrome in mice receiving a massive amount of radiation. J Radiat Res 51: 145–156. 19959877 10.1269/jrr.09078

[29] Frolen H, Luning KG, Ronnback C (1961) The effect of x-irradiation on various mouse strains due to their genetic background. I. Lethality after acute irradiation. Radiat Res 14: 381–393. 13702205

[30] Grahn D (1958) Acute Radiation Response of Mice from a Cross between Radiosensitive and Radioresistant Strains. Genetics 43: 835–843. 17247799 10.1093/genetics/43.5.835 PMC1209923

[31] Grahn D, Hamilton KF (1957) Genetic Variation in the Acute Lethal Response of Four Inbred Mouse Strains to Whole Body X-Irradiation. Genetics 42: 189–198. 17247690 10.1093/genetics/42.3.189 PMC1209824

[32] Kallman RF, Kohn HI (1956) The influence of strain on acute x-ray lethality in the mouse. I. LD50 and death rate studies. Radiat Res 5: 309–317. 13370831

[33] Paris F, Fuks Z, Kang A, Capodieci P, Juan G, et al (2001) Endothelial apoptosis as the primary lesion initiating intestinal radiation damage in mice. Science 293: 293–297. 11452123 10.1126/science.1060191

[34] Polistena A, Johnson LB, Ohiami-Masseron S, Wittgren L, Back S, et al (2008) Local radiotherapy of exposed murine small bowel: apoptosis and inflammation. BMC Surg 8: 1. 18173838 10.1186/1471-2482-8-1 PMC2248567

[35] Zheng H, Wang J, Koteliansky VE, Gotwals PJ, Hauer-Jensen M (2000) Recombinant soluble transforming growth factor beta type II receptor ameliorates radiation enteropathy in mice. Gastroenterology 119: 1286–1296. 11054386 10.1053/gast.2000.19282

[36] Helmrath MA, VanderKolk WE, Can G, Erwin CR, Warner BW (1996) Intestinal adaptation following massive small bowel resection in the mouse. J Am Coll Surg 183: 441–449. 8912612

[37] Otterson MF (2007) Effects of radiation upon gastrointestinal motility. World J Gastroenterol 13: 2684–2692. 17569136 10.3748/wjg.v13.i19.2684 PMC4147116

[38] Summers RW, Flatt AJ, Prihoda MJ, Mitros FA (1987) Effect of irradiation on morphology and motility of canine small intestine. Dig Dis Sci 32: 1402–1410. 3691278 10.1007/BF01296667

