# Supplementary figures and images for: Localized Intestinal Radiation and Liquid Diet Enhance Survival and Permit Evaluation of Long-Term Intestinal Responses to High Dose Radiation in Mice
Source: PLoS One. 2012 Dec 7;7(12):e51310. doi: 10.1371/journal.pone.0051310 (PMC3517426; doi:10.1371/journal.pone.0051310)

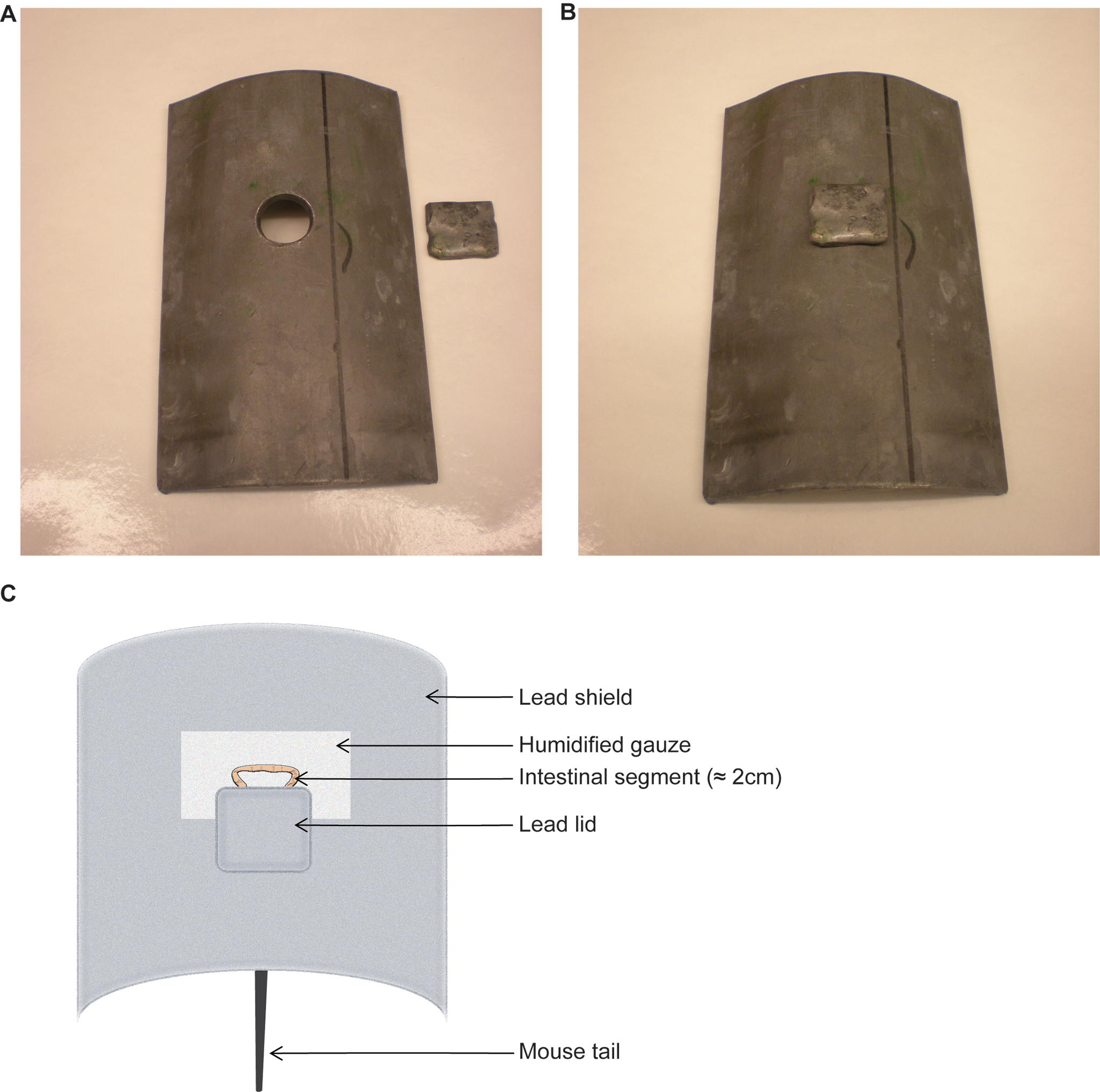

Supplement: Figure S1 — Custom-made lead shield. The lead shield was custom-designed so that it completely protects the mice from a 14 Gy radiation dose at a distance of 47 cm from the X-Ray source (320 kV/s, 10 mA; 2.8 Gy/min) equipped with a 2 mm Al filter. A. The opening in the center of the shield allows isolation and subsequent radiation of an exteriorized intestinal segment. B. The lead lid ensures complete cover of the mouse body during radiation of the exteriorized intestinal segment. C. The schematic illustrates the positioning of the mouse under the lead shield, the exteriorized segment on a humidified gauze and the lead lid covering the opening of the shield. (TIF) [file pone.0051310.s001.tif]

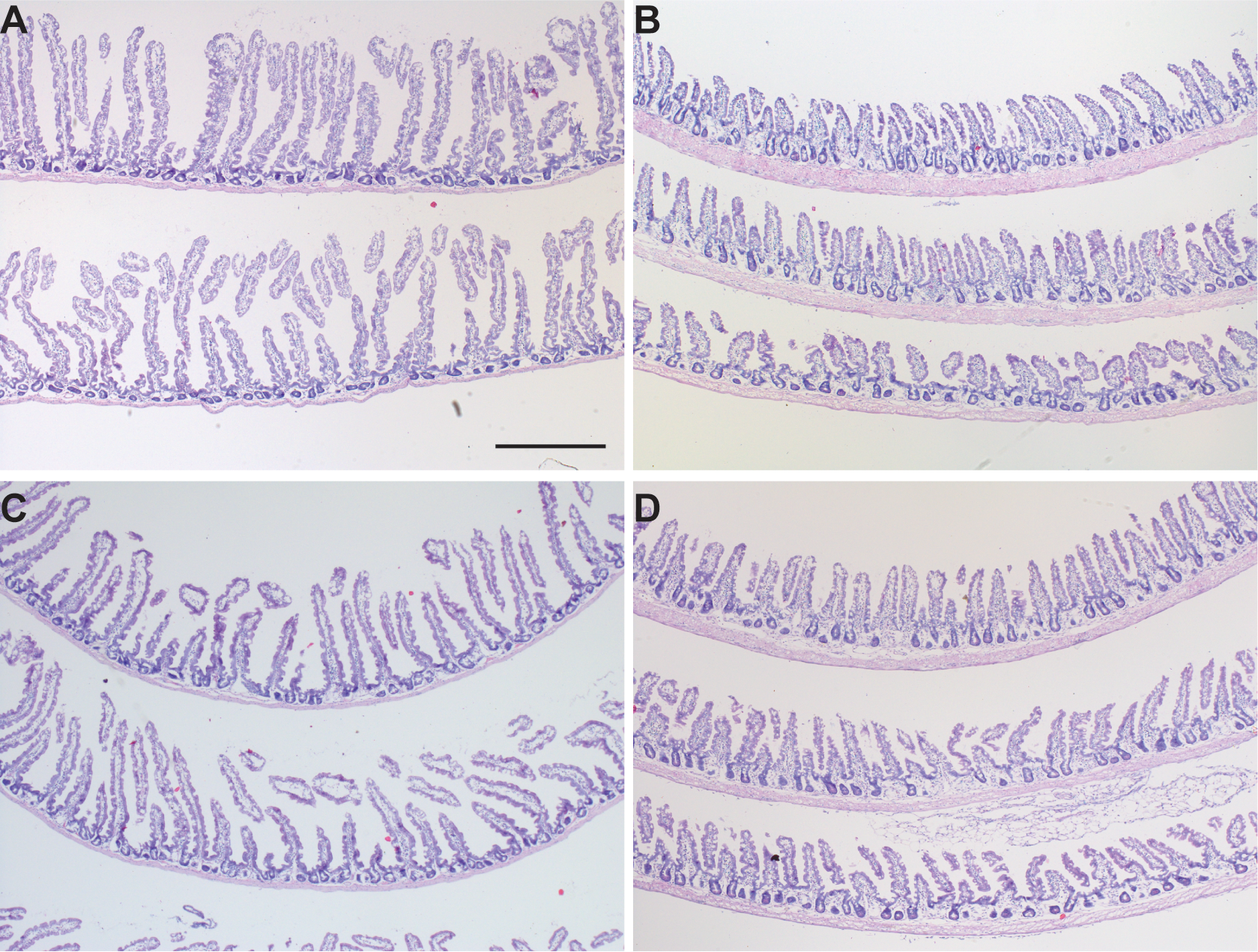

Supplement: Figure S2 — Duodenum and ileum of sham controls and segment-radiated mice show no epithelial damage. Illustrative photographs of H&E-stained Swiss-Rolled sections of duodenum and ileum of sham controls (A and B, respectively) demonstrate that the surgical procedure of the segment radiation method induces no damage to the intestinal epithelium (n = 3). Scale Bar: 500 µm. Similarly the duodenum (C) and the ileum (D) of mice radiated on a specific segment exhibited no epithelial damage, further attesting to the specificity of the segment radiation method. (TIF) [file pone.0051310.s002.tif]
